# Prosthetic joint infection due to Candida species: Case series and review of literature

**DOI:** 10.1097/MD.0000000000019735

**Published:** 2020-04-10

**Authors:** Eduardo Schincariol Saconi, Vladimir Cordeiro de Carvalho, Priscila Rosalba Domingos de Oliveira, Ana Lúcia Lei Munhoz Lima

**Affiliations:** Instituto de Ortopedia e Traumatologia, Hospital das Clíncias HCFMUSP, Faculdade de Medicina, Universidade de São Paulo, SP, Brazil.

**Keywords:** *Candida*, diagnosis, prosthetic joint infection, treatment

## Abstract

**Introduction:**

The increase in the number of patients with prosthetic joints will entail a rise in the absolute number of infections associated with these procedures. Although less frequent, infections by *Candida* species are also expected to increase, and the clinical and surgical management of these cases is based on case reports and opinion of specialists. The objective of the present study was to review the available literature and describe the cases of prosthetic joint infection caused by *Candida* species in patients of the Institute of Orthopedics and Trauma of the University of São Paulo Faculty of Medicine Clinics Hospital (IOT-HCFMUSP) between 2007 and 2014.

**Patient concerns:**

Eleven patients were diagnosed with prosthetic joint infection due to *Candida* with mean age of 65 years. The most frequent comorbidities were heart disease and diabetes mellitus, and the main personal antecedent was previous bacterial infection in the prosthetic joint. At least one risk factor for fungal infection was present in 73% of the patients. There was no difference between the prevalence of infections caused by *Candida albicans* and non-albicans *Candida* species, and there was bacterial co-infection in 55% of the cases.

**Diagnosis:**

For building up the case series, patients with cultures of bone and joint specimens that were positive for *Candida* species and had a clinical diagnosis of prosthetic joint infection were included in the case series.

**Interventions:**

Surgical debridement with removal of the prosthesis was the most frequently used surgical approach (45%). All patients were treated with monotherapy, and the most frequently used antifungal agent was fluconazole. The total duration of antifungal therapy was 6 months in 73% of the cases.

**Outcomes:**

After the initial management, 73% of the patients achieved clinical remission.

**Conclusion:**

The most indicated initial management was debridement with removal of the prosthesis, and the most used treatment regimen was fluconazole monotherapy. The most prevalent treatment duration was 6 months. The initial management led to a favorable outcome in 73% of the cases.

**Descriptors:**

Prosthetic joint infection, *Candida*, treatment, and diagnosis.

## 1 Introduction

The number of patients with joint prostheses has been increasing in recent decades, and 500,000 primary hip arthroplasties and over 3 million knee arthroplasties will be performed by 2030.^[1]^ Infection is expected to occur in 1% to 5% of these procedures, with the most frequently causative agents being Gram-positive bacteria, followed by Gram-negative bacteria and fungi.^2^,^3^

Fungal infections probably account for 0.6% to 1.4% of the total number of infected arthroplasties,^4^,^5^,^6^,^7^,^8^ with *Candida* species being the most frequent agents.^9^,^10^,^11^ Because of the low occurrence of infections caused by fungal agents, there are no published clinical trials that address the questions regarding the clinical and surgical management of these cases. The main international guidelines concerning this topic are based on case reports and opinion of specialists.^12^,^13^,^14^,^15^

Therefore, case reports of infections caused by fungal agents, especially by *Candida* species, play a very important role in improving the understanding of the affected population and of the initially adopted approaches that effectively lead to remission or cure of infection.

## 2 Method

This research is registered under protocol number 50713015.2.0000.0068 that can be found in www.plataformabrasil.saude.gov.br.

This is a retrospective, single-center, non-consecutive case series report. The identification of the cases of infection caused by *Candida* species in prosthetic joints in patients of the Institute of Orthopedics and Trauma of the University of São Paulo Faculty of Medicine Clinics Hospital (IOT-HCFMUSP) was performed by searching and intersecting the following data from electronic databases: bone and joint specimen cultures positive for *Candida* species between 2007 and 2014, patients who underwent an arthroplasty between 2006 and 2014, and patients with a diagnosis of postoperative infection between 2006 and 2014 (ICD 10: T84.1 and T84.5).

The study included patients with available cultures of tissues of the bone-joint system that were aseptically collected, were positive for *Candida* species, and who had a diagnosis of prosthetic joint infection made by the medical team. Patients have provided informed consent for publication of the case.

After selection of patients, their medical records were evaluated searching for predefined data such as gender, age, comorbidities, risk factor for fungal infection, clinical and surgical aspects, and antifungal treatment. The considered outcomes were remission, when there were no signs and symptoms of infection, and relapse, when there were.

To detect the cases reported in the literature, a search was performed in the electronic databases PubMed, Embase, Web of Knowledge, and LILACS from the earliest records of the databases until March 31, 2016, using the terms (“prosthesis related infection” OR “shoulder infection” OR “elbow infection” OR “knee infection” OR “hip infection” OR “ankle infection”) AND (“mycosis” OR “candida”).

## 3 Results

Eleven cases of infection caused by *Candida* species were detected in the prosthetic joints of patients of the IOT-HCFMUSP between 2007 and 2014. Of these, 6 patients were women (55%) and 5 patients were men (45%). The mean age was 65.1 years (range, 52–85 years) (Table 1).

**Table 1:**
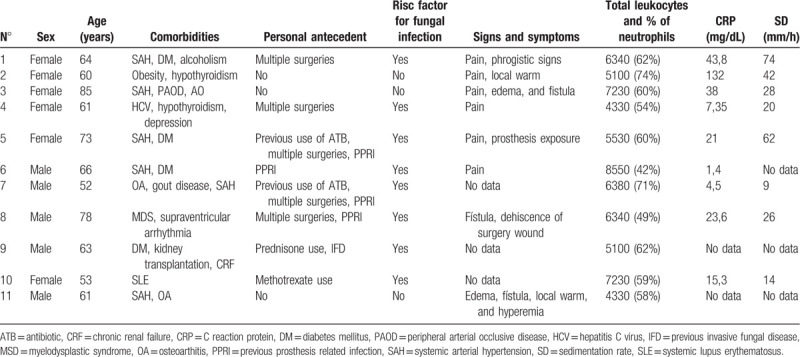
Demographic data, comorbidities, personal antecedent, signs and symptoms, and laboratorial tests of patients with prosthesis related infections due to *Candida* species.

The comorbidities and personal clinical antecedents described in Table 1 show that the most prevalent comorbidity was heart disease (7/11), followed by diabetes mellitus (4/11). The most prevalent clinical antecedents were previous bacterial infections in the concerned prosthetic joint and multiple procedures in the joint, with 4 occurrences each. At least one risk factor for fungal infection was present in 73% of the cases.

Six patients had hip prostheses and five patients had knee prostheses, and the primary indication for joint prosthesis implantation was osteoarthritis (Table 2).

**Table 2:**
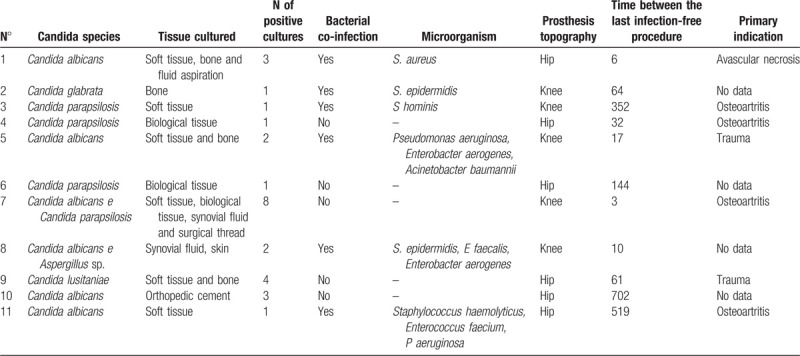
Microbiological and prosthesis related aspects of patients with prosthesis related infections due to *Candida* species.

The signs and symptoms and the nonspecific laboratory tests are described in detail in Table 1. The analysis of these data showed that pain and fistula formation were the most reported clinical findings and that no patient had leukocytosis (mean C-reactive protein level [CRP] of 31.2 mg/dL and mean sedimentation rate [SR] of 34.8 mm/h).

Table 2 shows that *Candida albicans* and non-albicans *Candida* infections were equally prevalent. There was an association between the 2 *Candida* species in 1 patient (patient no. 7) and between *Candida* and *Aspergillus* in another patient (patient no. 8).

Bacterial co-infection was detected in 6 cases (55%). It was caused by Gram-positive cocci in 3 cases and by an association between Gram-negative bacilli and Gram-positive cocci in another 3 cases.

The mean time between the last infection-free procedure and diagnosis of infection caused by a *Candida* species was 173.7 weeks (median, 61 weeks).

The most indicated initial approach was surgical debridement with removal of the prosthesis (5/11), followed by one-stage exchange (4/11) (Table 3). The time between debridement and two-stage exchange arthroplasty was 5.3 weeks. In the latter case, an antifungal agent was not added to the orthopedic cement used in the surgical procedure.

**Table 3:**
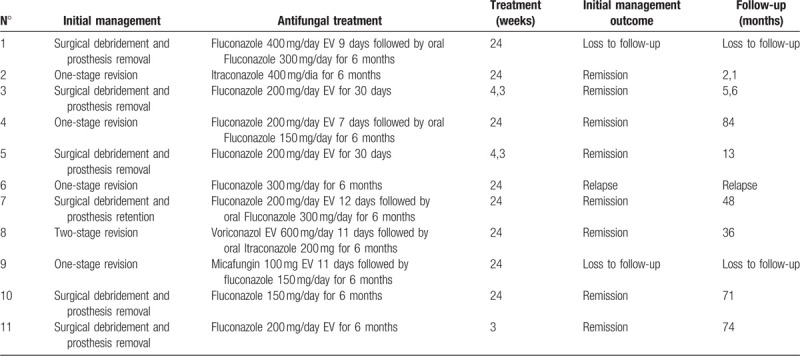
Initial management and antifungal treatment of patients with prosthesis related infections due to *Candida* species.

Monotherapy was used in all cases, and an intravenous antifungal agent was used in 8 patients; in 5 of these cases, there was a subsequent switch to an orally administered antifungal agent to end the treatment. The most frequently used antifungal agent was fluconazole, at doses that varied between 150 and 400 mg/day. The detailed description of the antifungal agents is shown in Table 3.

The treatment duration was 24 weeks for 8 patients and varied between 3 and 4.3 weeks for the other 3 patients.

The outcomes of the described cases are shown in Table 3, where it can be seen that the initial management led to remission in 8 cases (73%). There were 2 losses to follow-up and one recurrence with indication for surgical debridement and prosthesis removal. Amputation to control the infection was necessary in 1 patient (patient no. 5). Patient no. 8 had an infection by *Pseudomonas aeruginosa* in the same area after treatment of the infection caused by *C albicans* and *Aspergillus* spp. and was given suppressive chronic treatment with ciprofloxacin. Patient no. 11 had an infection by *P aeruginosa*, *Acinetobacter baumannii*, *Staphylococcus haemolyticus*, and *Bacteroides* in the same area after treatment of the infection caused by *C albicans*; the treatment in this case consisted of serial surgical debridement and specific antibacterial treatment, which resulted in remission of the infection.

The follow-up time of patients in remission was more than 1 year in 6 cases.

After literature review, 45 published articles were included and most of them were case reports or case series. A total of 94 cases of fungal infection were selected and analyzed.

## 4 Discussion

The majority of cases of prosthetic joint infection caused by *Candida* species occurred in women, which is in line with other published series^7^,^10^,^16^,^17^; however, there are also reports of male sex predominance.^8^,^9^,^18^

The mean age at diagnosis was 63 years, a result that is similar to those obtained in other studies^8^,^10^; however, there are reports of diagnosis being made in higher age groups also.^7^,^11^,^16^,^17^,^18^

The risk factors for fungal infection are the following: diabetes mellitus, chronic kidney disease, cancer, use of immunosuppressants, rheumatic diseases, prolonged use of antimicrobial agents, multiple procedures, and previous prosthetic joint infection.^10^,^15^,^19^

At least one risk factor was present in 73% of the patients included in this series. This percentage was also high in the largest published reports (varying between 69% and 100% of the patients).^7^,^8^,^10^,^16^,^18^

Pain was reported by 75% of the patients in this series, and edema was exhibited by 25% of them. Wang et al^[17]^ and Azzam et al^[10]^ reported that these symptoms were present in all patients included in their studies. Fistula formation occurred in 37.5% of the patients in the present series and has been described by other authors as varying between 57% and 80%.^7^,^17^,^20^

The patients did not have leukocytosis, which is in accordance with data published by other authors.^11^,^18^ The mean CRP level was 31.2 mg/dL, and it was within the normal range in 2 cases. However, Hwang et al^[11]^ and Azzam et al^[10]^ reported means of 4.3 and 17.5 mg/dL, respectively, and the occurrence of normal values in some patients. Normal results were also reported by Wang et al.^[17]^ The mean SR in the present study was 34.8 mm/h, and it was within the normal range in 2 cases. However, Hwang et al^[11]^ and Azzam et al^[10]^ reported means of 39 and 54 mm/h with some patients also having normal values.

In the present series, *C albicans* and non-albicans *Candida* were equally prevalent. Klatte et al^[18]^ and Anagnostakos et al^[9]^ also reported case series with equal distribution of these infections; however, there is a divergence between studies that report the predominance of either *C albicans* ^8^,^10^,^16^ or non-albicans *Candida*.^11^,^17^

Bacterial co-infection occurred in 55% of the cases in this series. This percentage is similar to that reported by Ueng et al.^[8]^ In the latter, all cases were caused by Gram-positive cocci, whereas associated Gram-negative bacilli were detected in half of the cases of the present study. Hwang et al^[11]^ and Azzam et al^[10]^ reported the presence of co-infection in 19% and 16% of the cases, respectively.

Infections occurred up to 1 year after the last infection-free procedure in 27% of the cases, which is in line with other publications.^8^,^18^ By contrast, all cases in the series published by Dutronc et al^[7]^ were diagnosed up to 1 year after the placement of the prosthesis. Time between the implantation of the prosthesis and diagnosis of infection may be related to the pathophysiological mechanism of infection acquisition.

Hip prosthesis infection was the most prevalent in the present series, a finding that was also shown in studies published by other authors^9^,^16^,^18^; however, there are reports which show that the knee was the most affected area.^7^,^8^,^10^

Removal of the prosthesis with subsequent revision surgery is the procedure of choice of some authors,^8^,^9^,^10^,^11^ with remission rates varying between 89% and 100%^8^,^11^,^17^; however, Klatte et al^[18]^ reported that one-stage revision had a remission rate of 90% and could be considered as a safe procedure that resulted in remission.

In the present case series, there were 4 cases of remission and 1 loss to follow-up among the 5 patients who underwent surgical debridement and prosthesis removal. There were 2 cases of remission, 1 case of recurrence, and 1 loss to follow-up among the 4 patients who underwent one-stage revision. The patients who underwent two-stage revision and surgical debridement with prosthesis retention also achieved clinical remission. The patient who underwent debridement and prosthesis retention received the diagnosis of prosthetic joint infection 3 weeks after the primary arthroplasty, which allowed an attempt to treat the infection while retaining the prosthesis.

The combined use of antifungal agents in the treatment of these infections has been widely described^7^,^16^,^18^,^21^,^22^,^23^,^24^,^25^,^26^,^27^,^28^,^29^,^30^,^31^,^32^; however, this strategy was not used in the present series.

According to Pappas et al,^[13]^ the use of fluconazole and echinocandins is strongly recommended for the treatment of joint infections caused by *Candida* species, whereas Parvizi et al^[12]^ stated that fluconazole or any amphotericin B product is indicated for this type of infection.

All patients included in this series received azoles for the treatment of the infection caused by *Candida* species. The use of both azoles^7^,^8^,^9^,^16^,^17^,^18^,^23^,^24^,^25^,^27^,^32^,^33^,^34^,^35^,^36^,^37^,^38^,^39^,^40^,^41^,^42^,^43^,^44^ and amphotericin B^22^,^26^,^28^,^29^,^30^,^31^,^45^,^46^,^47^,^48^,^49^,^50^,^51^,^52^,^53^,^54^ is widely described in the literature. The publications that report amphotericin B as the primary antifungal agent are mostly from the 1980s and 1990s, whereas those that report the use of azoles have been published since the 2000s.

The treatment duration for fungal infections related to prosthetic joints should vary according to the classification of the infections as acute or chronic, but the current guidelines do not address this issue. Pappas et al^[13]^ suggest a treatment duration between 6 and 12 months in cases of chronic osteomyelitis and of 6 weeks in cases of septic arthritis, whereas Parvizi et al^[12]^ proposed a treatment duration of 6 weeks.

Ueng et al^[8]^ found a positive association between a longer use of antifungal agents and treatment success. In the present series, 8 patients received treatment for 6 months and only 1 of them experienced recurrence of the infection, whereas the 3 patients who were treated between 3 and 4.3 weeks had the prosthesis removed and progressed to remission.

Remission was achieved in 73% of the cases of the present series. Two patients were lost to follow-up, and two cases had a follow-up of <1 year. The rate of remission after the initial management described in the main published case series varied between 0% and 100%.^11^,^16^,^17^

Amputation to control the infection was necessary in 1 case of this case series. Dutronc et al^[7]^ reported the need for amputation in 16% of the cases, and sporadic cases have also been reported in the literature.^37^,^50^

The ideal follow-up time to determine the cure of joint infections has not been well defined in the literature. In the present case series, 6 cases had a follow-up time of more than 1 year and 2 cases were followed for a shorter period. Follow-up times longer^8^,^11^,^17^,^18^ and shorter^9^,^16^ than 1 year have been reported in the literature.

This is one of the largest single-center case series of prosthetic joint infection due to *Candida* species and the limitations of these study are: the initial surgical approach and antifungal and dose selection varied among cases, follow up time were <1 year in two cases and there were two losses of follow-up.

Considering that prosthetic joint infection due to *Candida* species are rare, prospective multicenter studies are needed to provide better evidence to support clinical decisions.

## Author contributions

**Conceptualization:** Eduardo Schincariol Saconi, Vladimir Cordeiro de Carvalho, Priscila Rosalba Domingos de Oliveira, Ana Lúcia Lei Munhoz Lima.

**Data curation:** Eduardo Schincariol Saconi.

**Formal analysis:** Eduardo Schincariol Saconi.

**Methodology:** Eduardo Schincariol Saconi.

**Supervision:** Ana Lúcia Lei Munhoz Lima.

**Writing – original draft:** Eduardo Schincariol Saconi.

**Writing – review & editing:** Vladimir Cordeiro de Carvalho, Priscila Rosalba Domingos de Oliveira, Ana Lúcia Lei Munhoz Lima.

Eduardo Schincariol Saconi: 0000-0002-0584-4347.
